# Childhood trauma and non-suicidal self-injury among Chinese students: the mediating role of depression and the moderating role of perceived teacher legitimacy

**DOI:** 10.3389/fpsyg.2026.1780390

**Published:** 2026-06-30

**Authors:** Yongjian Lin, Weiwei Sun, Hongji Liu, Yahan Liu, Xiaoxiao Xin, Jing Qian

**Affiliations:** 1 Wenzhou Seventh People’s Hospital, Wenzhou Medical University, Wenzhou, China; 2 College of Education, Wenzhou University, Wenzhou, China; 3 Wenzhou Research Center for Family Tradition and Family Education, Wenzhou, China; 4 Shandong Yingcai University, Jinan, China; 5 Qingdao Jimo District Shibei Middle School, Qingdao, China; 6 The Third Affiliated Hospital of Wenzhou Medical University, Wenzhou, China

**Keywords:** childhood trauma, depression, moderated mediation, non-suicidal self-injury, perceived teacher legitimacy

## Abstract

**Background:**

Childhood trauma is a risk factor for non-suicidal self-injury (NSSI), but school-based factors in this pathway remain unclear. This study examined whether depression indirectly linked childhood trauma to NSSI and whether perceived teacher legitimacy moderated the trauma–depression association.

**Methods:**

A cross-sectional survey was conducted in 2025 with 576 students from two senior high schools and two universities in northern and southern mainland China. The sample exceeded the minimum size estimated using the standard cross-sectional survey formula, *n* = *Z*^2^*p*(1 − *p*)/*d*^2^. Childhood trauma, depression, perceived teacher legitimacy, and NSSI were measured using standardized self-report questionnaires. Gender, grade level, and parental educational attainment were controlled. Pearson correlations and PROCESS Model 7 with 5,000 bootstrap resamples were used.

**Results:**

Childhood trauma was positively associated with depression and NSSI. Depression partially mediated the association between childhood trauma and NSSI. Perceived teacher legitimacy moderated the trauma–depression association; unexpectedly, this association was stronger at higher levels of perceived teacher legitimacy.

**Conclusion:**

Depression may be one important, but not exclusive, pathway linking childhood trauma to NSSI. Perceived teacher legitimacy appears to have context-dependent effects rather than being uniformly protective. These cross-sectional findings support the need for trauma-informed school support, early screening for depression and NSSI risk, and longitudinal research.

## 1 Introduction

Childhood trauma refers to abuse, neglect, or other harmful experiences occurring within relationships of responsibility, trust, or power that result in, or have the potential to result in, harm to a child’s health, survival, development, or dignity (World Health Organization, 2024; Massullo et al., 2023). Epidemiological evidence indicates that the prevalence of childhood trauma is substantial among university student populations; systematic reviews report that Chinese undergraduates retrospectively disclose appreciable rates of childhood maltreatment (Fu et al., 2018). A large body of research shows that such early adversities increase the risk of psychological maladjustment in adolescence and young adulthood, including elevated perceived stress, depressive symptomatology, and anxiety (Bhattarai et al., 2023; Li et al., 2025).

In recent years, non-suicidal self-injury (NSSI) has been recognized as a major mental health concern among adolescents and university students. NSSI often functions as a maladaptive strategy for regulating or relieving negative affect and is closely associated with suicidal ideation and suicide attempts (Apicella et al., 2025; Escofet-Colet et al., 2025; Ye et al., 2022). Evidence from China indicates substantial prevalence of NSSI in adolescent and student samples and robust associations between childhood trauma and later self-injurious behavior (Cheng et al., 2023; Sun et al., 2023). Prior research further suggests that the link from childhood trauma to NSSI is often transmitted via proximal mechanisms such as depressive symptoms, impaired emotion recognition, and deficits in emotion regulation (Tatnell et al., 2017; Calvete et al., 2022). Because schools and teachers constitute central socialization contexts, teacher–student interactions characterized by procedural justice features, including respectful treatment, equitable practices, and procedural transparency, may shape students’ emotional responses and behavioral adaptation and thus either mitigate or amplify the effects of childhood trauma (Tyler and Trinkner, 2018). Clarifying the mechanisms through which childhood trauma relates to NSSI among students is therefore both an important empirical challenge and a practical priority for school-based prevention and intervention.

### 1.1 Childhood trauma and non-suicidal self-injury

A growing body of recent research has documented a robust association between childhood trauma and non-suicidal self-injury (NSSI). Childhood trauma commonly includes emotional abuse, physical abuse, sexual abuse, emotional neglect, and physical neglect. Recent systematic and empirical studies indicate that these maltreatment experiences are linked to increased risk for NSSI during adolescence and early adulthood (Calvo et al., 2024; Gong and Zhang, 2024; Yang et al., 2025). Mechanistic accounts suggest that early maltreatment disrupts the development of adaptive emotion regulation, fosters persistent negative self-schemas such as self-criticism and shame, and undermines interpersonal trust. These vulnerabilities increase the likelihood that individuals will use NSSI as a short-term strategy for affect regulation or as a form of self-directed punishment (Nock, 2010; Yates, 2004). Evidence from Chinese samples parallels international findings, indicating that higher levels of childhood trauma are associated with increased prevalence of NSSI among middle school and university students (Cheng et al., 2023; Lin et al., 2020). Collectively, these findings position childhood trauma as a distal risk factor for NSSI whose effects are transmitted through more proximal psychological processes.

### 1.2 Depression as a mediating mechanism

Depression, characterized by low mood, anhedonia, and negative self-referential cognition, has been widely linked to NSSI (Guan et al., 2024; Marshall et al., 2013). From a developmental psychopathology perspective, childhood trauma may increase vulnerability to depressive symptoms by undermining emotion regulation, self-worth, and interpersonal security. Depressive symptoms, in turn, may increase psychological pain and self-punitive tendencies, making NSSI more likely as a maladaptive strategy for regulating or externalizing distress (Calvete et al., 2022; Qu et al., 2023).

However, depression should not be treated as the sole or primary mechanism linking childhood trauma to NSSI. Depression is a broad syndrome, and more proximal processes within or related to depression, such as rumination, hopelessness, shame, alexithymia, and emotion regulation difficulties, may be more directly involved in self-injurious behavior. Recent evidence also suggests that rumination may mediate the association between childhood trauma and self-injury among depressed adolescents (Zhou et al., 2025). Therefore, the present study examines depression as one possible affective pathway between childhood trauma and NSSI, while recognizing that other cognitive and emotional mechanisms may operate in parallel.

### 1.3 Perceived teacher legitimacy as a contextual influence

Perceived teacher legitimacy refers to students’ perceptions of teachers as fair, rightful, and trustworthy authority figures. This construct is grounded in procedural justice and legal socialization theories, which suggest that respectful treatment, fair decision-making, and opportunities for voice strengthen perceptions of authority legitimacy and support norm internalization (Tyler, 2006a; Li et al., 2024). In school contexts, recent evidence shows that fair and supportive teacher–student relationships shape students’ moral norms, self-control, and later perceptions of authority legitimacy (Nivette et al., 2022). Perceiving teachers as procedurally just may also weaken the adverse influence of delinquent peers on students’ perceptions of school authority (Cardwell et al., 2021). As a contextual resource, perceived teacher legitimacy may therefore shape whether students with adverse early experiences view teachers as accessible sources of support or mainly as sources of normative expectation and evaluation.

### 1.4 The present study and hypotheses

Applied to the present model, teacher legitimacy may attenuate the extent to which childhood trauma translates into depressive symptoms by promoting help-seeking behavior, facilitating access to emotional and instrumental resources, and fostering a predictable and fair classroom climate. For students with trauma histories, perceived legitimacy may increase the likelihood that teachers are experienced as meaningful sources of support rather than solely as agents of normative pressure. On this basis, teacher legitimacy is conceptualized as a potential buffer in the pathway from childhood trauma to depression.

Drawing on the theoretical and empirical literature reviewed above, the present study tests the following hypotheses:

*H1*: Childhood trauma will be positively associated with non-suicidal self-injury among Chinese students.

*H2*: Depression will mediate the association between childhood trauma and non-suicidal self-injury.

*H3*: Teacher legitimacy will moderate the association between childhood trauma and depression, such that the positive relationship between childhood trauma and depressive symptoms will be weaker at higher levels of perceived teacher legitimacy.

## 2 Methods

### 2.1 Participants and procedure

Data were collected in 2025 using convenience sampling. Participants were recruited from two universities and two senior high schools in northern and southern mainland China. The survey was administered online via Wenjuanxing during regular class time. To standardize administration, the responsible researcher, who was also one of the authors, distributed the questionnaire link and read a scripted instruction sheet explaining the study purpose, confidentiality, voluntary participation, and response requirements. Only students who provided informed consent completed the questionnaire. A total of 576 valid responses were obtained.

Sample size adequacy was assessed using the standard formula for cross-sectional surveys, *n* = *Z*^2^*p*(1 − *p*)/*d*^2^ (Charan and Biswas, 2013). With *Z* = 1.96, *p* = 0.50, and *d* = 0.05, the minimum required sample size was 384. The final sample of 576 exceeded this requirement and was adequate for the planned regression-based mediation and moderation analyses. The questionnaire took approximately 15–20 min to complete, and participants received a small, randomly assigned monetary incentive. The study was approved by the School of Education Research Ethics Committee of Wenzhou University (approval no. WZUED20250401).

### 2.2 Measures

#### 2.2.1 Childhood Trauma Questionnaire–Short Form (CTQ-SF)

Childhood trauma was assessed using the Short Form of the Childhood Trauma Questionnaire (CTQ-SF), originally developed by Bernstein et al. (2003). The CTQ-SF is a retrospective self-report instrument designed to assess adverse experiences during childhood. It consists of 28 items, including 25 clinical items and three validity items, and covers five dimensions: emotional abuse, physical abuse, sexual abuse, emotional neglect, and physical neglect. Items are rated on a 5-point Likert scale ranging from 1 (never) to 5 (very often), yielding a total score ranging from 25 to 125, with higher scores indicating more severe childhood trauma. The Chinese version of the CTQ-SF was adapted and validated by Zhao et al. (2005). In the present study, the scale demonstrated good internal consistency, with a Cronbach’s alpha coefficient of 0.845. A sample item is “Someone in my family hit me so hard that I had to see a doctor or go to the hospital.”

#### 2.2.2 Assessment of non-suicidal self-injury

Based on the Chinese version of the Self-Injurious Thoughts and Behaviors Interview (SITBI) developed by Nock et al. (2007) and adapted to the purposes of the present study, non-suicidal self-injury (NSSI) was assessed by measuring seven common forms of self-injurious behavior, including cutting or piercing the skin to cause bleeding, deliberately pinching or scratching oneself, burning the skin, hitting oneself, intentionally banging one’s head against or punching objects, biting oneself, intentionally ingesting foreign objects or medication, and deliberately pulling one’s hair. A total of seven items were included. Each item was rated on a six-point scale, with the question framed as “In the past year, have you ever harmed yourself in the following ways?,” and response options ranging from 0 to 5 times. Higher frequencies indicated greater severity of non-suicidal self-injury. In the present study, the scale demonstrated excellent internal consistency (Cronbach’s *α* = 0.939).

#### 2.2.3 Ultra-brief depression screening scale

Depressive symptoms were measured using the depression subscale of the Patient Health Questionnaire-4 (PHQ-4), a widely used ultra-brief screening instrument validated and standardized in general populations by Löwe et al. (2010). The depression subscale consists of two items: “Feeling down, depressed, or hopeless” and “Little interest or pleasure in doing things.” Responses are rated on a 4-point scale ranging from 0 (not at all) to 3 (nearly every day), with higher total scores indicating more frequent depressive symptoms. In the present study, the depression subscale demonstrated good internal consistency, with a Cronbach’s alpha coefficient of 0.889.

#### 2.2.4 Perceived teacher legitimacy

Perceived teacher legitimacy was measured using a Chinese translation of an international teacher legitimacy scale. The scale assesses students’ perceptions of teachers as legitimate authority figures and their endorsement of voluntary compliance with teacher authority. It includes 10 items rated on a 5-point Likert scale from 1 (strongly disagree) to 5 (strongly agree), with higher scores indicating stronger perceived teacher legitimacy. A sample item is “Even if I do not like the way my teacher treats me, I should still follow the teacher’s rules.”

The scale was translated and back-translated by bilingual researchers, with discrepancies resolved through discussion. A small group of students reviewed the Chinese version to ensure clarity and contextual appropriateness. In this study, Cronbach’s *α* was 0.701.

### 2.3 Data analysis

All statistical analyses were conducted using SPSS version 26.0. First, Harman’s single-factor test was performed to assess potential common method bias. An unrotated exploratory factor analysis extracted nine factors with eigenvalues greater than 1, and the first factor accounted for 22.97% of the total variance, which is below the commonly used threshold of 40%, indicating that common method bias was not a serious concern in the present study.

Subsequently, descriptive statistics and Pearson correlation analyses were conducted to examine the basic relationships among the study variables. To test the moderated mediation model, Hayes (2018) PROCESS macro for SPSS was employed, specifically Model 7. A bootstrapping procedure with 5,000 resamples was used to estimate the indirect effect of childhood trauma on non-suicidal self-injury via depression, as well as the moderating effect of teacher legitimacy on the path from childhood trauma to depression.

## 3 Results

### 3.1 Descriptive statistics and correlation analyses

The analytic sample consisted of 576 students, including 390 males and 186 females. By grade, first-year students accounted for the largest proportion of the sample (44.4%), followed by second-year students (22.6%), third-year students (11.5%), and fourth-year students (11.3%). Most parents had attained secondary-school education or below, including 77.6% of fathers and 82.5% of mothers.

The means, standard deviations, and Pearson correlation coefficients for the main study variables are presented in Table 1. Childhood trauma was positively correlated with depression (*r* = 0.35, *p* < 0.001) and non-suicidal self-injury (NSSI; *r* = 0.38, *p* < 0.001), and negatively correlated with perceived teacher legitimacy (*r* = −0.30, *p* < 0.001). Depression was positively associated with NSSI (*r* = 0.40, *p* < 0.001) and negatively associated with perceived teacher legitimacy (*r* = −0.17, *p* < 0.001). The correlation between perceived teacher legitimacy and NSSI was not significant (*r* = −0.01, *p* = 0.845). Overall, these correlations were consistent with the proposed model and supported further mediation and moderation analyses.

**Table 1:** Descriptive statistics and correlation analysis of main variables (*N* = 576).

Variable	*M*	*SD*	1	2	3	4
1. Childhood trauma	36.04	10.47	1			
2. Depression	3.44	1.58	0.35^***^	1		
3. Non-suicidal self-injury	1.05	4.80	0.38^***^	0.40^***^	1	
4. Perceived teacher legitimacy	35.93	5.91	−0.30^***^	−0.17^***^	−0.01	

M = Mean, SD = Standard deviation; Pearson product–moment correlation coefficients are presented below the diagonal; ^***^p < 0.001.

### 3.2 Moderated mediation analysis

PROCESS Model 7 was used to test the moderated mediation model, with gender, grade level, paternal education, and maternal education included as covariates. As shown in Table 2, the model predicting depression was significant, *F* = 17.651, *p* < 0.001, explaining 17.9% of the variance. Childhood trauma positively predicted depression (*β* = 0.052, *p* < 0.001), and its interaction with perceived teacher legitimacy was significant (*β* = 0.002, *p* = 0.015), indicating first-stage moderation. The model predicting NSSI was also significant, *F* = 31.553, *p* < 0.001, explaining 25.0% of the variance. Depression positively predicted NSSI after controlling for childhood trauma (*β* = 0.834, *p* < 0.001), supporting its mediating role.

**Table 2:** Regression analysis of moderated mediation model.

Variable	Depression			Non-suicidal self-injury		
	*β*	se	*t*	*β*	se	*t*
Intercept	2.918	0.232	12.573^***^	2.553	0.748	3.412^**^
Childhood trauma	0.052	0.006	8.446^***^	0.124	0.018	6.928^***^
Depression	—	—	—	0.834	0.121	6.909^***^
Perceived teacher legitimacy	−0.017	0.011	−1.590	—	—	—
Childhood trauma × Legitimacy	0.002	0.001	2.441^*^	—	—	—
Gender	0.460	0.132	3.496^***^	1.125	0.384	2.929^**^
Grade	0.054	0.025	2.167^*^	0.199	0.073	2.731^**^
Paternal education	0.013	0.070	0.184	0.186	0.203	0.917
Maternal education	−0.146	0.076	−1.932	0.040	0.219	0.183
*R^2^*	0.179			0.250		
*F*	17.651^***^			31.553^***^		

*N* = 576; ^*^p < 0.05, ^**^p < 0.01, ^***^p < 0.001; interaction terms are mean-centered.

**Figure 1:**
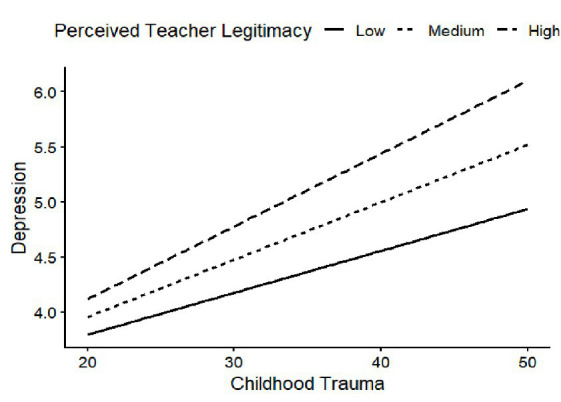
Simple slopes of childhood trauma predicting depression at low, mean, and high levels of perceived teacher legitimacy. Low and high levels of perceived teacher legitimacy represent one standard deviation below and above the mean, respectively.

Simple slope analyses showed that childhood trauma significantly predicted depression at low (*β* = 0.038, *p* < 0.001, 95% CI [0.023, 0.053]), mean (*β* = 0.052, *p* < 0.001, 95% CI [0.040, 0.064]), and high levels of perceived teacher legitimacy (*β* = 0.066, *p* < 0.001, 95% CI [0.048, 0.083]). The effect strengthened as perceived teacher legitimacy increased, suggesting that perceived teacher legitimacy amplified, rather than buffered, the association between childhood trauma and depression (Figure 1).

Conditional indirect effects were examined using a bootstrap procedure with 5,000 resamples, and the results are presented in Table 3. Across low, mean, and high levels of teacher legitimacy, the indirect effects of childhood trauma on non-suicidal self-injury via depression were all statistically significant, as the corresponding bootstrap confidence intervals did not include zero. However, the index of moderated mediation was 0.002, and its 95% bootstrap confidence interval [−0.0001, 0.004] included zero, indicating that the differences in the magnitude of the indirect effects across levels of teacher legitimacy did not reach statistical significance. In addition, both the direct effect and the total effect of childhood trauma on non-suicidal self-injury were significant. Taken together, these findings support a first-stage moderated mediation model in which teacher legitimacy moderates the association between childhood trauma and depression, but does not significantly alter the overall strength of the indirect effect through depression.

**Table 3:** Test of moderated mediation effects (bootstrap = 5,000).

Effect type	Effect value	Boot SE	Boot 95% CI
Conditional indirect effect			
Low teacher legitimacy (M − 1SD)	0.032	0.011	[0.013, 0.057]
Average teacher legitimacy (M)	0.043	0.013	[0.021, 0.071]
High teacher legitimacy (M + 1SD)	0.055	0.017	[0.025, 0.092]
Index of moderated mediation	0.002	0.001	[−0.0001, 0.004]
Direct effect (Childhood trauma → NSSI)	0.124	0.018	[0.089, 0.159]
Total effect	0.167	0.019	[0.130, 0.205]

## 4 Discussion

### 4.1 Discussion of the main findings

Using a moderated mediation framework, the present study examined the mechanisms and boundary conditions through which childhood trauma is associated with non-suicidal self-injury (NSSI). The findings showed a complex pattern: depression partially mediated the association between childhood trauma and NSSI, and the first stage of this indirect pathway, from childhood trauma to depression, was significantly moderated by perceived teacher legitimacy. Notably, the direction of this moderating effect was contrary to the original hypothesis.

Consistent with prior and recent evidence, childhood trauma showed both direct and indirect associations with NSSI. Recent studies have confirmed childhood maltreatment as a robust risk factor for adolescent NSSI (Calvo et al., 2024) and have further shown that depressive symptoms may mediate the association between childhood trauma and NSSI (Yang et al., 2025; Liu et al., 2025). Recent adolescent studies have also emphasized that NSSI is shaped by multiple predisposing and precipitating factors, including maladaptive coping, bullying, disappointment, and limited opportunities for emotional sharing or support (Iswanti et al., 2024; Kandar et al., 2025). Extending this literature, the present findings suggest that depression is one important pathway through which childhood trauma may be linked to self-injurious behavior. Childhood trauma may undermine emotion regulation, self-worth, and interpersonal security, thereby increasing vulnerability to depression. In turn, depressive states may increase psychological pain and make NSSI more likely as a maladaptive attempt to reduce or externalize intolerable negative affect (Calvete et al., 2022; Qu et al., 2023).

At the same time, the partial mediation effect indicates that depression does not fully explain the link between childhood trauma and NSSI. Other mechanisms may operate in parallel, including impulsivity, maladaptive cognitive schemas, alexithymia, stress perception, and interpersonal difficulties. Recent work has also shown that stress perception and teacher–student relationships may jointly shape the association between childhood trauma and NSSI among high school students (Liu et al., 2024). Together, these findings support an integrative view in which childhood trauma is associated with NSSI through multiple affective, cognitive, and relational pathways. Depression should therefore be understood as one important, but not exclusive, psychological pathway rather than the central mechanism linking childhood trauma to NSSI.

Although procedural justice theory usually regards authority legitimacy as a protective resource that promotes trust, normative internalization, and psychological adjustment (Tyler, 2006b; Tyler and Trinkner, 2018), the present findings show a more complex pattern. Among students with childhood trauma experiences, higher perceived teacher legitimacy strengthened, rather than weakened, the association between trauma and depression. This suggests that the psychological role of legitimacy may depend on students’ prior relational experiences. For trauma-exposed students, highly legitimate teachers may represent not only support and guidance, but also stronger standards, expectations, and evaluative pressure. Students who have experienced early blame, neglect, or punishment may be especially likely to interpret such expectations through self-blame and shame. When they fail to meet perceived authority standards, they may attribute setbacks to personal inadequacy, which can deepen depressive feelings (Tangney et al., 2014; Tilghman-Osborne et al., 2008).

Another possible explanation concerns help-seeking and emotional disclosure. Students who strongly endorse teacher authority may be reluctant to disclose family-related trauma or psychological distress, because they fear being judged, losing face, or disappointing teachers whom they respect. In this case, high teacher legitimacy does not necessarily mean high emotional safety. If legitimacy is experienced mainly as obedience, discipline, or moral expectation, vulnerable students may become more cautious about expressing distress. This may reduce opportunities for timely support and allow trauma-related negative emotions to accumulate. This interpretation is consistent with trauma-informed educational perspectives, which emphasize that teachers can support trauma-exposed students only when the school relationship is perceived as safe, confidential, and emotionally responsive (Maynard et al., 2019; Berger et al., 2025).

More broadly, recognizing an authority as legitimate does not always mean perceiving that authority as emotionally caring. When legitimacy is based mainly on procedural or institutional justification rather than relational support, its normative demands may outweigh its protective function (Bernburg and Thorlindsson, 2007; Gau and Brunson, 2010). This point is important because teacher legitimacy was measured as a unidimensional construct in the present study. Therefore, the findings should not be taken to mean that teacher authority is generally harmful for trauma-exposed students. Rather, they suggest that different components of teacher legitimacy may have different psychological meanings. Procedural fairness, rule enforcement, respect, relational warmth, and emotional accessibility may not operate in the same way. Recent cross-cultural studies suggest that teacher–student relationships are not culturally neutral. Their psychological meaning may vary across educational contexts, with Chinese settings placing relatively greater emphasis on harmony, respect, responsibility, and the moral role of teachers (Roorda et al., 2023; Xu et al., 2023). This interpretation is also consistent with recent legal socialization theory, which argues that, in China’s relationship-oriented cultural context, authority-related meanings are formed and reinforced through family, school, and peer networks, including teacher–student relations (Xu, 2026). In such contexts, perceived teacher legitimacy may therefore be linked not only to emotional support, but also to compliance, role obligations, and evaluative expectations. For students with relational trauma histories, these authority meanings may activate insecurity, self-criticism, and conflicted expectations toward adults, thereby amplifying depressive responses.

### 4.2 Implications for healthcare practice

The findings have practical implications for school-based healthcare and mental health services. Adolescents with childhood trauma histories should be prioritized for early NSSI risk screening, especially when depressive symptoms are present. Recent studies show that adolescent NSSI is related to multiple psychological and contextual risks, including maladaptive coping, bullying, disappointment, and limited opportunities for emotional sharing (Iswanti et al., 2024; Kandar et al., 2025). Thus, routine assessment of depression may help school nurses, counselors, and mental health professionals identify students who need timely support.

Intervention should focus not only on reducing self-injurious behavior, but also on the emotional distress that sustains it. Recent review evidence suggests that psychoeducation, emotion-regulation training, mindfulness-based strategies, and structured self-help tools may be useful components of NSSI prevention and intervention programs (Escofet-Colet et al., 2025). In practice, depression screening can be combined with brief emotion-regulation support, safety planning, and referral pathways for students with repeated or severe NSSI.

The finding regarding perceived teacher legitimacy also has implications for school support. Teachers may serve as important gatekeepers for identifying at-risk students, but authority alone does not ensure emotional safety. For trauma-exposed students, support should emphasize respectful communication, confidentiality, non-stigmatizing responses, and accessible help-seeking channels. Collaboration among teachers, school nurses, counselors, parents, and external mental health services may help build a more coordinated prevention system.

### 4.3 Limitations and future directions

A strength of this study is that it examined depression and perceived teacher legitimacy within an integrated moderated mediation framework linking childhood trauma to non-suicidal self-injury (NSSI). Several limitations should also be noted. First, all key variables were assessed using self-report questionnaires, and childhood trauma was measured retrospectively, which may have introduced recall bias and common method bias. Future studies could include parent, teacher, or peer reports to improve data credibility and ecological validity. Second, the cross-sectional design limits causal interpretation. The findings should therefore be understood as statistical associations rather than evidence of temporal or causal pathways. Longitudinal or multi-wave studies are needed to clarify the ordering and possible reciprocal relations among childhood trauma, depression, perceived teacher legitimacy, and NSSI. Third, perceived teacher legitimacy was measured using a Chinese translation of an international teacher legitimacy scale. Although translation, back-translation, and preliminary student review were conducted, further validation in Chinese student samples is needed, including factor structure, convergent and discriminant validity, criterion validity, and measurement invariance. More fine-grained measures should also distinguish procedural fairness, relational support, emotional accessibility, respect, and normative authority to identify which aspects are protective or potentially burdensome for trauma-exposed students. Finally, future research should examine more proximal mechanisms, such as impulsivity, alexithymia, rumination, and emotion regulation difficulties, and develop school-based prevention programs for adolescents exposed to childhood trauma.

## 5 Conclusion

This study suggests that depression may be one important psychological pathway linking childhood trauma to non-suicidal self-injury (NSSI) among Chinese students. The findings also indicate that perceived teacher legitimacy may not be uniformly protective; instead, higher perceived teacher legitimacy strengthened the association between childhood trauma and depressive symptoms. This pattern highlights the context-dependent role of authority legitimacy and suggests that, for students with trauma histories, highly legitimate authority may be associated with greater self-blame and emotional distress rather than reduced risk. Overall, the findings extend current understanding of NSSI by integrating individual vulnerability with school-based authority dynamics, and they suggest that procedural fairness should be combined with emotional accessibility, confidentiality, and trauma-informed support in school-based prevention and intervention efforts.

## Data Availability

The raw data supporting the conclusions of this article will be made available by the authors, without undue reservation.

## References

[ref1] Apicella M. Pontillo M. Maglio G. Di Vincenzo C. Della Santa G. Andracchio E. . (2025). Non-suicidal self-injury in adolescents: a clinician's guide to understanding the phenomenon, diagnostic challenges, and evidence-based treatments. Front. Psych. 16:1605508. doi: 10.3389/fpsyt.2025.1605508, 40800631 PMC12339462

[ref2] Berger E. Marston N. Batsilas G. M. O’Donohue K. Holford T. Allen K.-A. . (2025). What can teachers do to help young people exposed to traumatic events? Young peoples’ perspectives. Int. J. Educ. Res. 134:102839. doi: 10.1016/j.ijer.2025.102839

[ref3] Bernburg J. G. Thorlindsson T. (2007). Community structure and adolescent delinquency in Iceland: a contextual analysis. Criminology 45, 415–444. doi: 10.1111/j.1745-9125.2007.00083.x

[ref4] Bernstein D. P. Stein J. A. Newcomb M. D. Walker E. Pogge D. Ahluvalia T. . (2003). Development and validation of a brief screening version of the childhood trauma questionnaire. Child Abuse Negl. 27, 169–190. doi: 10.1016/S0145-2134(02)00541-0, 12615092

[ref5] Bhattarai A. King N. Adhikari K. Dimitropoulos G. Devoe D. Byun J. . (2023). Childhood adversity and mental health outcomes among university students: a longitudinal study. Can. J. Psychiatr. 68, 510–520. doi: 10.1177/07067437221111368, 36000272 PMC10408556

[ref6] Calvete E. Royuela-Colomer E. Maruottolo C. (2022). Emotion dysregulation and mindfulness in non-suicidal self-injury. Psychiatry Res. 314:114691. doi: 10.1016/j.psychres.2022.114691 35777277

[ref7] Calvo N. Lugo-Marín J. Oriol M. Pérez-Galbarro C. Restoy D. Ramos-Quiroga J.-A. . (2024). Childhood maltreatment and non-suicidal self-injury in adolescent population: a systematic review and meta-analysis. Child Abuse Negl. 157:107048. doi: 10.1016/j.chiabu.2024.107048, 39332140

[ref8] Cardwell S. Mazerolle L. Luengen K. Piquero A. (2021). Perceiving teachers as procedurally just limits the negative effects of delinquent peer associations: an analysis of Australian adolescent boys’ and girls’ perceptions of school authority. J. Soc. Issues 77, 547–576. doi: 10.1111/josi.12447

[ref9003] Charan J. Biswas T. (2013). How to calculate sample size for different study designs in medical research? Indian J. Psychol. Med. 35, 121–126. doi: 10.4103/0253-7176.116232 24049221 PMC3775042

[ref9] Cheng F. Shi L. W. Wang S. J. Jin Q. Xie H. B. Wang B. N. . (2023). The relationship between childhood traumatic experience and suicidal tendency in non-suicidal self-injury behavior patients. BMC Psychiatry 23:401. doi: 10.1186/s12888-023-04863-0, 37277735 PMC10240761

[ref10] Escofet-Colet I. Casadó-Marín L. C. Orós-Navas L. Raventós-Torner R. (2025). Non-suicidal self-injury in adolescents: a systematic review on prevention and intervention programmes. J. Child Adolesc. Psychiatr. Nurs. 38:e70039. doi: 10.1111/jcap.70039, 41055224 PMC12502457

[ref11] Fu H. L. Feng T. J. Qin J. B. Wang T. T. Wu X. B. Cai Y. M. . (2018). Reported prevalence of childhood maltreatment among Chinese college students: a systematic review and meta-analysis. PLoS One 13:e0205808. doi: 10.1371/journal.pone.0205808, 30321243 PMC6188789

[ref12] Gau J. M. Brunson R. K. (2010). Procedural justice and order maintenance policing: a study of inner-city young men’s perceptions of police legitimacy. Justice Q. 27, 255–279. doi: 10.1080/07418820902763889

[ref13] Gong X. Zhang L. L. (2024). Childhood maltreatment and non-suicidal self-injury in adolescents: testing a moderated mediating model. J. Interpers. Violence 39, 925–948. doi: 10.1177/08862605231197747, 38229266

[ref9002] Hayes A. F. (2018). Introduction to mediation, moderation, and conditional process analysis: A regression-based approach (2nd ed.). New York, NY: Guilford Press.

[ref14] Guan M. Z. Liu J. C. Li X. H. Cai M. Bi J. Zhou P. . (2024). The impact of depressive and anxious symptoms on non-suicidal self-injury behavior in adolescents: a network analysis. BMC Psychiatry 24:229. doi: 10.1186/s12888-024-05599-1, 38532354 PMC10967160

[ref15] Iswanti D. I. Lai L. L. Saifudin I. M. M. Y. Kandar K. Dewi R. K. Cahyaningrum D. D. (2024). The predictor of non-suicidal self-injury behavior among adolescents: a cross-sectional study. J. Ners 19, 125–133. doi: 10.20473/jn.v19i2.54610

[ref16] Kandar Dewi R. K. Cahyaningrum D. D. Wimala D. Anggriani H. Luberingsih T. A. . (2025). Predisposition and precipitation factors of non-suicidal self-injury behaviors among adolescents. Front. Nurs. 12, 271–278. doi: 10.2478/fon-2025-0029

[ref18] Li J. C. M. Zhang S. Y. Sun I. Y. Ho A. S. K. (2024). Police legitimacy and procedural justice for children and youth: a scoping review of definitions, determinants, and consequences. Front. Sociol. 9:1409080. doi: 10.3389/fsoc.2024.1409080, 39385980 PMC11461445

[ref19] Li M. Zhang J. H. Xu T. Xu Z. S. (2025). Childhood trauma and psychological distress among college freshmen: the mediating role of sense of security and moderating effect of friendship quality. Stud. Psychol. Behav. 23, 82–90. doi: 10.12139/j.1672-0628.2025.01.011

[ref20] Lin L. H. Gan M. X. Guo Z. B. Zhang B. Y. Jiang Q. (2020). Psychological abuse and neglect and non-suicidal self-injury in adolescents: a mediated moderating model. Chin. J. Clin. Psychol. 28, 1140–1143. doi: 10.16128/j.cnki.1005-3611.2020.06.012

[ref21] Liu Y. L. Fang Y. Chen Y. L. Qin F. Y. Li X. R. Feng R. B. . (2024). Relationship between childhood trauma and non-suicidal self-injury in high school students: the mediating role of the stress perception and the moderating role of teacher-student relationship. BMC Psychol. 12:379. doi: 10.1186/s40359-024-01883-7, 38978110 PMC11232308

[ref22] Liu A. N. Zhang R. H. N. Yang S. W. Luo Y. Wang Z. Y. Peng C. . (2025). The mediation of depressive symptoms between different types of childhood maltreatment and non-suicidal self-injury. Sci. Rep. 15:15270. doi: 10.1038/s41598-025-99601-9, 40312445 PMC12046006

[ref23] Löwe B. Wahl I. Rose M. Spitzer C. Glaesmer H. Wingenfeld K. . (2010). A 4-item measure of depression and anxiety: validation and standardization of the patient health Questionnaire-4 (PHQ-4) in the general population. J. Affect. Disord. 122, 86–95. doi: 10.1016/j.jad.2009.06.019, 19616305

[ref24] Marshall S. K. Tilton-Weaver L. C. Stattin H. (2013). Non-suicidal self-injury and depressive symptoms during middle adolescence: a longitudinal analysis. J. Youth Adolesc. 42, 1234–1242. doi: 10.1007/s10964-013-9919-3, 23371004

[ref25] Massullo C. De Rossi E. Carbone G. A. Imperatori C. Ardito R. B. Adenzato M. . (2023). Child maltreatment, abuse, and neglect: an umbrella review of their prevalence and definitions. Clin. Neuropsychiatry 20, 72–99. doi: 10.36131/cnfioritieditore20230201, 37250758 PMC10211430

[ref26] Maynard B. R. Farina A. Dell N. A. Kelly M. S. (2019). Effects of trauma-informed approaches in schools: a systematic review. Campbell Syst. Rev. 15:e1018. doi: 10.1002/cl2.1018, 37131480 PMC8356508

[ref27] Nivette A. Obsuth I. Ribeaud D. Eisner M. (2022). Fair teachers, fair police? Assessing the pathways between perceptions of teacher and police authority in childhood and adolescence. J. Youth Adolescence 51, 193–207. doi: 10.1007/s10964-021-01537-6, 34783955 PMC8828593

[ref9001] Nock M. K. Holmberg E. B. Photos V. I. Michel B. D. (2007). Self-injurious thoughts and behaviors interview: Development, reliability, and validity in an adolescent sample. Psy. Assess. 19, 309–317. doi: 10.1037/1040-3590.19.3.309 17845122

[ref28] Nock M. K. (2010). Self-injury. Annu. Rev. Clin. Psychol. 6, 339–363. doi: 10.1146/annurev.clinpsy.121208.131258, 20192787

[ref29] Qu D. Y. Wen X. Liu B. W. Zhang X. He Y. H. Chen D. Y. . (2023). Non-suicidal self-injury in Chinese population: a scoping review of prevalence, method, risk factors and preventive interventions. Lancet Reg. Health West. Pac. 37:100794. doi: 10.1016/j.lanwpc.2023.100794, 37693882 PMC10485683

[ref30] Roorda D. Chen M. D. Zee M. (2023). “Affective student–teacher relationships and students’ engagement: a cross–cultural comparison of China and the Netherlands,” in Effective Teaching around the World: Theoretical, Empirical, Methodological and Practical Insights, eds. Maulana R. Helms-Lorenz M. Klassen R. M. (Cham: Springer International Publishing), 423–437.

[ref31] Sun Q. Li Y. L. Ye L. X. Si X. Y. (2023). Research progress on risk factors and pathogenesis of NSSI among adolescents. J. New Med. 54, 17–21. doi: 10.3969/j.issn.0253-9802.2023.01.004

[ref32] Tangney J. P. Stuewig J. Martinez A. G. (2014). Two faces of shame: the roles of shame and guilt in predicting recidivism. Psychol. Sci. 25, 799–805. doi: 10.1177/0956797613508790, 24395738 PMC4105017

[ref33] Tatnell R. Hasking P. Newman L. Taffe J. Martin G. (2017). Attachment, emotion regulation, childhood abuse and assault: examining predictors of NSSI among adolescents. Arch. Suicide Res. 21, 610–620. doi: 10.1080/13811118.2016.1246267, 27726519

[ref34] Tilghman-Osborne C. Cole D. A. Felton J. W. Ciesla J. A. (2008). Relation of guilt, shame, behavioral and characterological self-blame to depressive symptoms in adolescents over time. J. Soc. Clin. Psychol. 27, 809–842. doi: 10.1521/jscp.2008.27.8.809, 25419043 PMC4238306

[ref35] Tyler T. R. (2006a). Psychological perspectives on legitimacy and legitimation. Annu. Rev. Psychol. 57, 375–400. doi: 10.1146/annurev.psych.57.102904.190038, 16318600

[ref36] Tyler T. R. (2006b). Why People Obey the Law. Princeton: Princeton University Press.

[ref37] Tyler T. R. Trinkner R. (2018). Why Children Follow Rules: Legal Socialization and the Development of Legitimacy. Oxford: Oxford University Press.

[ref38] World Health Organization (2024). Child maltreatment. Available online at: https://www.who.int/news-room/fact-sheets/detail/child-maltreatment (Accessed May 29, 2026).

[ref39] Xu S. H. (2026). The three-dimensional dynamic model of legal socialization: a cross-cultural theoretical integration of Chinese and Western research. Leg. Criminol. Psychol. 31, 1–26. doi: 10.1111/lcrp.70028

[ref40] Xu C. M. Huizinga M. de Luca G. Pollé S. Liang R. W. Sankalaite S. . (2023). Cultural universality and specificity of teacher-student relationship: a qualitative study in Belgian, Chinese, and Italian primary school teachers. Front. Psychol. 14:1287511. doi: 10.3389/fpsyg.2023.1287511, 38034285 PMC10682107

[ref41] Yang L. H. Du X. Y. Huang M. X. (2025). Childhood maltreatment and non-suicidal self-injury: the mediating role of mentalization and depression. Eur. J. Psychotraumatol. 16:2466279. doi: 10.1080/20008066.2025.2466279, 39995338 PMC11864010

[ref42] Yates T. M. (2004). The developmental psychopathology of self-injurious behavior: compensatory regulation in posttraumatic adaptation. Clin. Psychol. Rev. 24, 35–74. doi: 10.1016/j.cpr.2003.10.001, 14992806

[ref43] Ye Z. Y. Xiong F. Li W. T. (2022). A meta-analysis of co-occurrence of non-suicidal self-injury and suicide attempt: implications for clinical intervention and future diagnosis. Front. Psych. 13:976217. doi: 10.3389/fpsyt.2022.976217, 36032240 PMC9411747

[ref44] Zhao X. Zhang Y. Li L. Zhou Y. Li H. Yang S. (2005). Reliability and validity of the Chinese version of the childhood trauma questionnaire. Chin. J. Clin. Rehabil. 9, 105–107.

[ref45] Zhou M. L. Wang S. J. Zheng D. D. Li J. Y. Yang Y. (2025). Gender-differentiated pathways from childhood trauma to self-injury: rumination as a mediator in depressed adolescents. BMC Psychol. 13:1057. doi: 10.1186/s40359-025-03440-2, 41013801 PMC12465670

